# Guanxin V Acts as an Antioxidant in Ventricular Remodeling

**DOI:** 10.3389/fcvm.2021.778005

**Published:** 2022-01-04

**Authors:** Bo Liang, Rui Li, Yi Liang, Ning Gu

**Affiliations:** ^1^Nanjing University of Chinese Medicine, Nanjing, China; ^2^Southwest Medical University, Luzhou, China; ^3^Nanjing Hospital of Chinese Medicine Affiliated to Nanjing University of Chinese Medicine, Nanjing, China

**Keywords:** Guanxin V, ventricular remodeling, oxidative stress, network pharmacology, virtual screening, molecular docking, two GXV pathways, validation

## Abstract

**Background:** Our previous studies have shown that Guanxin V (GXV) is safe and effective in the treatment of ventricular remodeling (VR), but its mechanism related to oxidative stress has not been studied deeply.

**Methods:** We applied integrating virtual screening and network pharmacology strategy to obtain the GXV-, VR-, and oxidative stress-related targets at first, and then highlighted the shared targets. We built the networks and conducted enrichment analysis. Finally, the main results were validated by molecular docking and solid experiments.

**Results:** We obtained 251, 11,425, and 9,727 GXV-, VR-, and oxidative stress-related targets, respectively. GXV-component-target-VR and protein–protein interaction networks showed the potential mechanism of GXV in the treatment of VR. The following enrichment analysis results gathered many biological processes and “two GXV pathways” of oxidative stress-related to VR. All our main results were validated by molecular docking and solid experiments.

**Conclusion:** GXV could be prescribed for VR through the mechanism, including complex interactions between related components and targets, as predicted by virtual screening and network pharmacology and validated by molecular docking and solid experiments. Our study promotes the explanation of the biological mechanism of GXV for VR.

## Introduction

Ventricular remodeling (VR) refers to the changes at the cellular and anatomical levels that occur based on gene expression changes, mainly cardiomyocytes, non-cardiomyocytes, and extracellular matrix (1). Cell structure and function are reconstructed and finally related to arrhythmia and heart failure, which can lead to death (2). Early reversal of VR has a significant effect on reducing major adverse cardiovascular events. Nowadays, the drugs used to treat VR are mainly empirical, including angiotensin-converting enzyme inhibitor/angiotensin receptor blocker/angiotensin receptor-neprilysin inhibitor, beta-blockers, and aldosterone receptor inhibitors. Nondrug therapy, such as device-assisted therapy, cannot benefit many patients because of its high price and high requirements for technology of the surgeons. Therefore, we urgently need an alternative and complementary therapy.

Traditional Chinese medicine, mainly from the East, has been used clinically for nearly 3,000 years and has gradually received acceptance and recognition abroad and overseas in the recent years (3, 4). Guanxin V (GXV) is a mixture of traditional Chinese medication, which has been used clinically for decades. Our previous clinical studies showed that GXV could increase ejection fraction, cardiac output, and stroke volume, left ventricular end-diastolic diameter, left ventricular end-systolic diameter, and left ventricular late diastolic A peak velocity while decreasing left ventricular early diastolic E peak velocity among coronary artery disease patients with VR with no adverse reaction, indicating that GXV is a potentially safe and effective treatment for VR (5). Moreover, our subsequent animal experiments also showed that GXV could inhibit or even reverse VR in animals with acute myocardial infarction (6).

**Figure 1:**
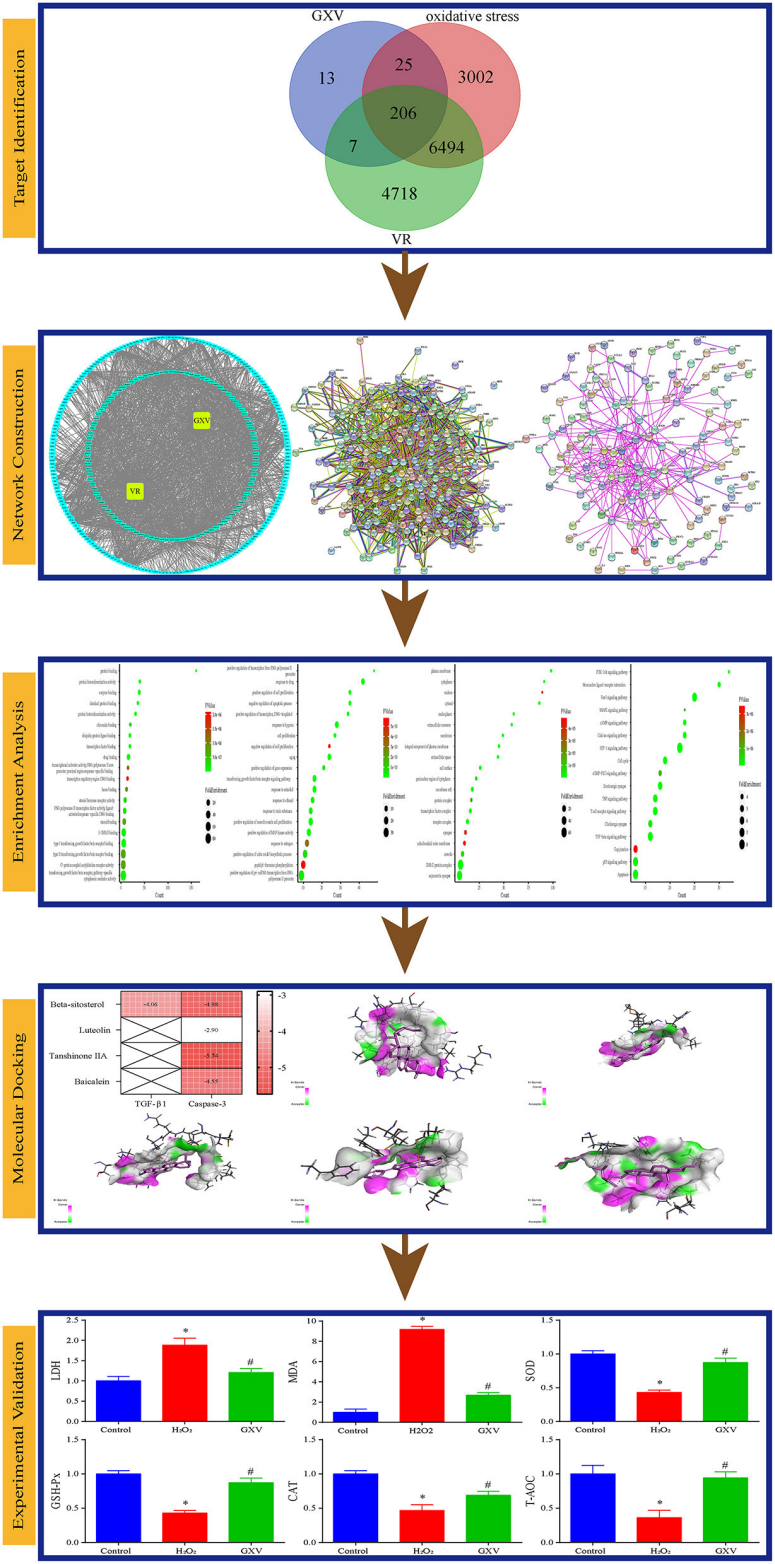
The overall workflow of this study.

Oxidative stress is the result of the imbalance between reactive oxygen species formation and enzymatic and non-enzymatic antioxidants (7). Excessive reactive oxygen species can cause oxidative damage to lipids, proteins, and DNA (8), and is accompanied by a significant decrease in antioxidant levels and antioxidant enzyme activities (9). Therefore, increased reactive oxygen species production or impaired antioxidant system could tilt the cell redox balance to oxidative imbalance and lead to the overproduction of reactive oxygen species (10). Oxidative stress is considered to be an important component of various diseases (11), including VR (12). In recent years, the study of the mechanism of oxidative stress in VR has achieved certain results (13, 14), showing that oxidative stress may be one of the targets of anti-VR (15, 16). Our previous studies have shown that GXV can reverse VR, but its effect on oxidative stress is not particularly clear. Here, we used virtual screening and network pharmacology methods to determine the oxidative stress-related targets of GXV in the treatment of VR, and further verify it through molecular docking and robust experiments (Figure 1). We hope that our results can further promote our understanding of the molecular biological mechanism of anti-oxidative stress of GXV in VR, and lay an experimental foundation for the further clinical application of GXV.

## Methods

### Targets Screening

We first obtained active compounds and corresponding targets for GXV from our previous study (17). In another previous study (6), we identified the effective components in GXV by ultra-performance liquid chromatography-quadrupole time-of-flight high-resolution mass spectrometry (UPLC-Q-TOF/HRMS E). In this study, we supplemented the targets of these effective components in SwissTargetPrediction by the structure of each effective component (18), as described previously (19). The targets of VR were obtained from our previous study (17). Taking “oxidative stress” as a keyword, we obtained oxidative stress-related targets from GeneCards, a searchable and integrative database that provides comprehensive information on all annotated and predicted human genes (20).

### Network Construction

All targets were standardized in Universal Protein (UniProt) (21), and then taking the intersection of the targets in GXV, VR, and oxidative stress, these shared targets were considered as key therapeutic targets related to GXV against oxidative stress of VR, as described previously (22), and were visualized by Venn diagram. We also constructed a GXV-component-target-VR (G-C-T-V) network to visualize the relationships of targets between GXV and VR. Moreover, the cytoHubba plugin (23) in Cytoscape (24) was applied to evaluate the multiple centralities. A protein–protein interaction (PPI) network of all shared targets was then constructed with all genes as the background (25). The network diagram was completed in the string database (26) with the organism option was set to *Homo sapiens* (Human) and medium confidence was more than 0.400. Then, we used the MCODE plugin (27) in Cytoscape (24) to cluster the PPI network based on the topology to find densely connected regions.

### Functional Enrichment

DAVID Bioinformatics Resources (28) was utilized to conduct functional enrichment analysis {including Gene Ontology [GO] terms (29) and Kyoto Encyclopedia of Genes and Genomes [KEGG] pathways (30)} of all shared targets. We also used *Homo sapiens* (Human) as the background. To avoid over counting duplicated genes, based on corresponding DAVID gene IDs, we calculated the Fisher Exact statistics to remove all redundancies in original IDs. The threshold of EASE score, a modified Fisher Exact *P* value, was applied for the evaluation of gene-enrichment analysis. Fisher Exact *P* value range from 0 to 1, ≤0.05 was considered strongly enriched, and equal to 0 were considered perfect enrichment. All results had to pass the thresholds (EASE ≤ 0.05 and the number of genes annotated with a GO term or KEGG pathway was more than or equal to 5) to ensure only significant enrichment terms were displayed. The top 20 results were visualized by the *ggplot2* package in R (31). KEGG Mapper is a collection of tools for KEGG mapping, which could realize the visualization of the situation of the certain gene set in the corresponding signaling pathways or other high-level enrichment features (32). We used this tool to mapper specific signaling pathways.

In addition, the ClueGO plugin (33) in Cytoscape (24) was used to create and visualize a functionally grouped network of terms in cluster networks from the PPI network.

### Computational Validation

Refer to our previous research results (17), we selected two key targets from established “two GXV pathways” (TGF-β1 and Caspase-3) and their corresponding compounds from the established G-C-T-V network (MOL000358 [Beta-Sitosterol], MOL000006 [Luteolin], MOL007154 [Tanshinone IIA], MOL002714 [Baicalein]) to validate computationally by molecular docking. Detailly, we used receptor-ligand molecular docking to assess these interactions. The structures of protein crystal and compound were obtained from Protein Data Bank (34) and PubChem (35), respectively. The binding energy was calculated by AutoDockTools (36) and the docking was visualized by Discovery Studio.

### Experimental Validation

H9c2(2-1) cells were incubated with H_2_O_2_ (300 μM) for 24 h to establish the oxidative stress model and then treated with GXV (1 g/L) for another 24 h (17). Supernatants and cells were harvested and the antioxidant activities were investigated *via* malondialdehyde (MDA, NJJCBIO, Nanjing, China), superoxide dismutase (SOD, mlbio, Shanghai Enzyme-linked Biotechnology Co., Ltd., Shanghai, China), lactate dehydrogenase (LDH, NJJCBIO, Nanjing, China), catalase (CAT, Jiangsu Meimian Industrial Co., Ltd., Yancheng, China), total antioxidant capacity (T-AOC, Beyotime, Shanghai, China), and glutathione peroxidase (GSH-PX, NJJCBIO, Nanjing, China) according to the instructions of the manufacturers.

## Results

### Identification of the Targets of GXV, VR, and Oxidative Stress

We obtained 119 active compounds and corresponding 196 targets for GXV from our previous study (17). Moreover, we supplemented 77 targets for 15 effective components in GXV. After integrating all the targets, we obtained 251 GXV-related targets. We got 11,425 known therapeutic targets for VR from our previous study (17). A total of 9,727 targets were identified as stress-related oxidative.

### Network Construction

**Figure 2:**
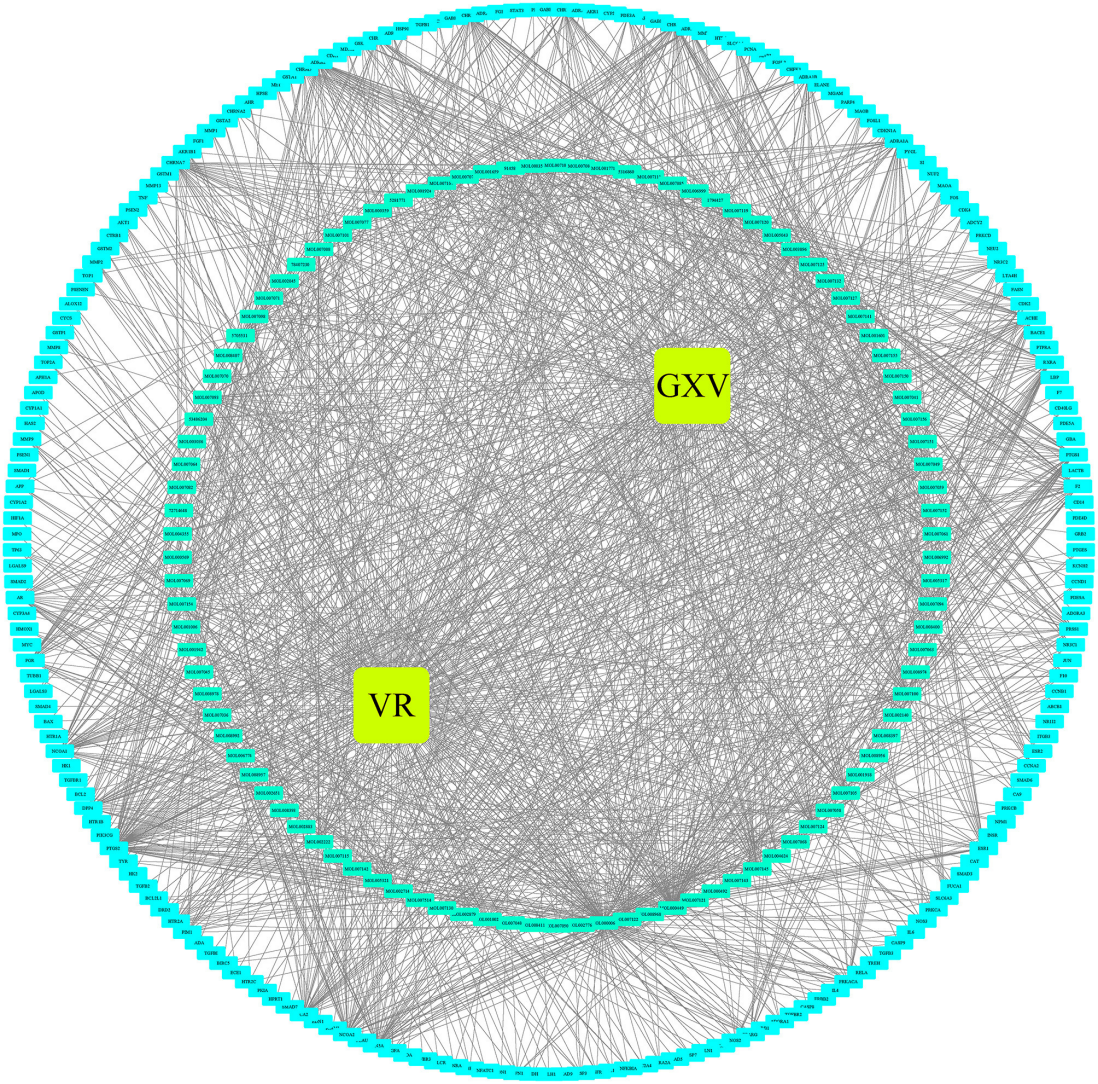
The G-C-T-V network.

The Venn diagram showed that there were 206 shared targets among 251 GXV-related targets, 11,425 VR-related targets, and 9,727 oxidative stress-related targets (Supplementary Figure S1). These 206 shared targets were considered as key therapeutic targets related to GXV against oxidative stress of VR. Subsequently, we built the G-C-T-V network to illustrate the potential mechanism of GXV acting on VR. To simplify the network, we only used the shared targets and their related compounds to construct the G-C-T-V network, which is composed of 314 nodes and 1,503 edges (Figure 2). Moreover, we revealed the most important nodes in this network. Multiple centralities demonstrated that MOL000006 (Luteolin), MOL003896 (7-Methoxy-2-Methyl Isoflavone), MOL007154 (Tanshinone IIA), 5705531 (Dihydrocatalpol), MOL007100 (Dihydrotanshinlactone), MOL000358 (Beta-Sitosterol), and MOL002714 (Baicalein) were the most important compounds in our G-C-T-V network (Table 1); while PTGS2, NCOA1, SCN5A, ADRB2, NCOA2, PTGS1, CHRM1, CHRNA7, F2, RXRA, ACHE, and CA2 were the most important targets in our G-C-T-V network (Table 2). We have reasons to believe that these compounds and targets played key roles in GXV for treating VR.

**Table 1:** Multiple centralities of compounds in the G-C-T-V network (MCC ≥ 30).

**Node**	**Annotation**	**Local-based method**				**Global-based method**						
		**MCC**	**DMNC**	**MNC**	**Degree**	**EPC**	**Bottle Neck**	**EcCentricity**	**Closeness**	**Radiality**	**Betweenness**	**Stress**
MOL000006	Luteolin	55	0	1	109	22.320	1	0.33333	158.0000	3.69649	9101.1310	192292
MOL003896	7-Methoxy-2-Methyl Isoflavone	38	0	1	38	20.399	1	0.33333	146.6667	3.58786	1058.2190	51582
MOL007154	Tanshinone IIA	38	0	1	38	17.247	2	0.33333	146.6667	3.58786	2350.5920	46280
5705531	Dihydrocatalpol	32	0	1	32	7.3510	24	0.33333	142.6667	3.54952	10983.5200	209800
MOL007100	Dihydrotanshinlactone	32	0	1	32	19.210	1	0.33333	142.6667	3.54952	618.3010	43020
MOL000358	Beta-Sitosterol	32	0	1	32	16.302	1	0.33333	142.6667	3.54952	1458.1340	37480
MOL002714	Baicalein	32	0	1	32	12.111	1	0.33333	142.6667	3.54952	1900.3660	30926

*MCC, maximal clique centrality; DMNC, density of maximum neighborhood Component; MNC, maximum neighborhood component; EPC, edge percolated component*.

**Table 2:** Multiple centralities of targets in G-C-T-V network (MCC ≥ 30).

**Node**	**Annotation**	**Local-based method**				**Global-based method**						
		**MCC**	**DMNC**	**MNC**	**Degree**	**EPC**	**Bottle Neck**	**Ec Centricity**	**Closeness**	**Radiality**	**Betweenness**	**Stress**
PTGS2	Prostaglandin-Endoperoxide Synthase 2	62	0	1	66	27.172	1	0.25	170.4167	3.82428	4497.1270	148412
NCOA1	Nuclear Receptor Coactivator 1	36	0	1	39	21.072	1	0.25	153.0833	3.65815	1082.7710	52242
SCN5A	Sodium Voltage-Gated Channel A Subunit 5	36	0	1	38	22.735	1	0.25	153.0833	3.65815	879.1755	49214
ADRB2	Adrenoceptor B 2	35	0	1	38	22.861	1	0.25	152.4167	3.65176	724.7611	48948
NCOA2	Nuclear Receptor Coactivator 2	33	0	1	38	18.692	1	0.25	151.0833	3.63898	2341.6570	62710
PTGS1	Prostaglandin-Endoperoxide Synthase 1	33	0	1	36	21.744	1	0.25	151.0833	3.63898	1046.6570	59386
CHRM1	Cholinergic Receptor Muscarinic 1	32	0	1	35	21.879	1	0.25	150.4167	3.63259	633.7786	43362
CHRNA7	Cholinergic Receptor Nicotinic A 7 Subunit	31	0	1	34	21.611	1	0.25	149.7500	3.62620	534.4842	40906
F2	Coagulation Factor II, Thrombin	31	0	1	32	16.676	1	0.25	149.7500	3.62620	853.0783	29594
RXRA	Retinoid X Receptor A	30	0	1	33	19.255	1	0.25	149.0833	3.61981	647.0734	40368
ACHE	Acetylcholinesterase	30	0	1	31	16.274	1	0.25	149.0833	3.61981	873.2213	28376
CA2	Carbonic Anhydrase 2	30	0	1	31	17.575	15	0.25	155.0833	3.77316	2965.7050	65226

*MCC, maximal clique centrality; DMNC, density of maximum neighborhood component; MNC, maximum neighborhood component; EPC, edge percolated component*.

**Figure 3:**
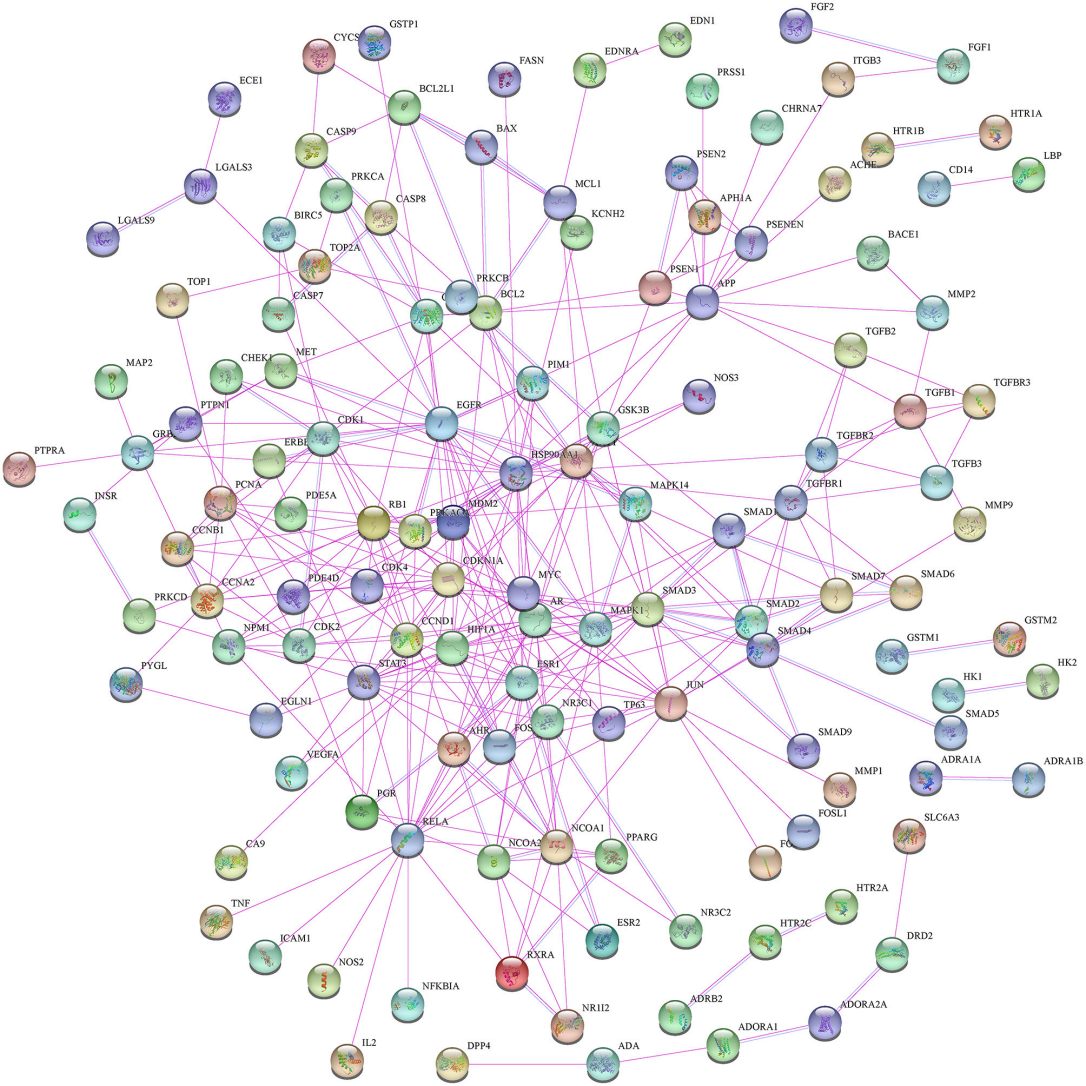
The protein–protein interaction (PPI) network with known interactions comes from experimentally determined evidence.

**Figure 4:**
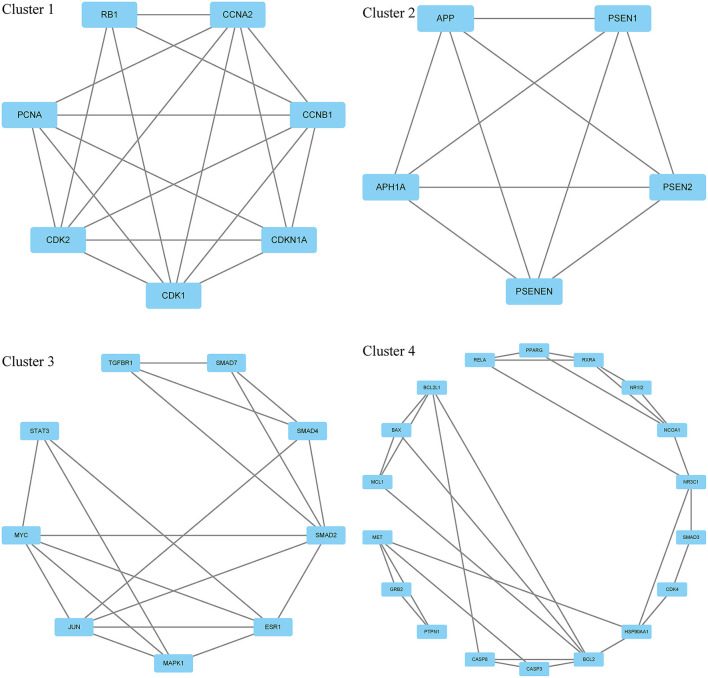
Four cluster networks are divided from the PPI network.

Moreover, in this study, we also constructed a PPI network of the 206 shared targets, which consisted of 206 nodes and 6,116 edges (Supplementary Figure S2). This means that the proteins have more interactions among themselves than a random set of proteins of similar size drawn from the genome. Such an enrichment indicates that the proteins are at least partially biologically connected as a group and the network highlights the complexity of interactions among proteins. We later applied the PPI network with known interactions supported by the experimentally determined evidence hiding disconnected nodes in the PPI network (Figure 3). Through the MCODE plugin, we obtained 4 cluster networks with the highest clustering scores (Table 3, Figure 4).

**Table 3:** Four cluster networks with the highest clustering scores.

**Cluster**	**Score**	**Nodes**	**Edges**	**Node IDs**
1	6.333	7	38	CDK2, CDKN1A, PCNA, RB1, CDK1, CCNA2, CCNB1
2	5.000	5	20	PSEN1, APP, PSEN2, PSENEN, APH1A
3	4.750	9	38	SMAD2, MYC, SMAD4, TGFBR1, SMAD7, MAPK1, STAT3, ESR1, JUN
4	3.412	18	58	GRB2, RXRA, CDK4, MCL1, HSP90AA1, MET, BAX, NCOA1, BCL2, BCL2L1, PTPN1, CASP3, PPARG, CASP8, RELA, SMAD3, NR1I2, NR3C1

### Functional Enrichment Analysis

**Figure 5:**
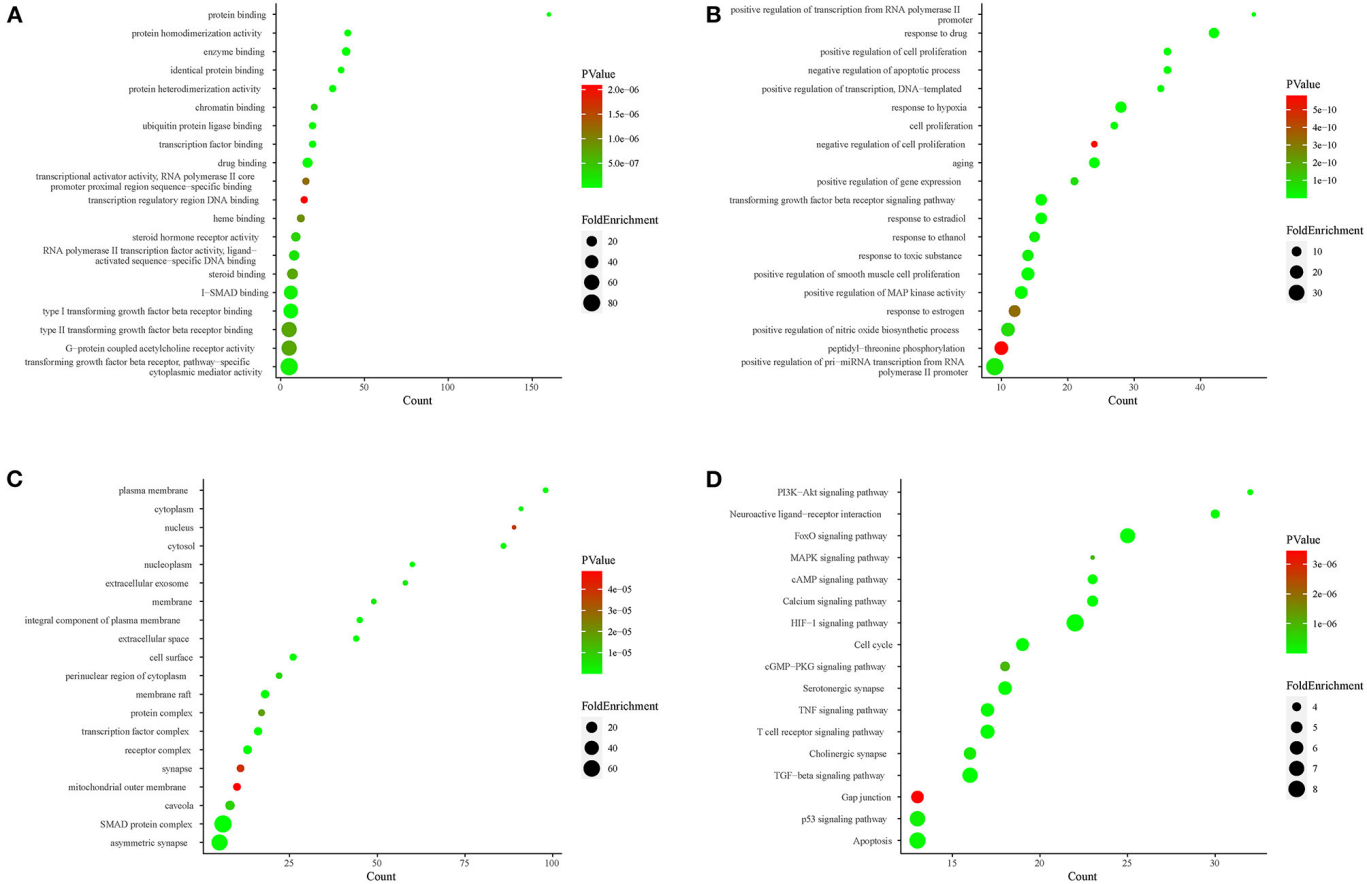
Functional enrichment analysis. **(A)** Top 20 MF of GO analysis colored by *P* value. **(B)** Top 20 BP of GO analysis colored by *P* value. **(C)** Top 20 CC of GO analysis colored by *P* value. **(D)** Kyoto Encyclopedia of Genes and Genomes (KEGG) pathway enrichment analysis colored by *P* value. The X-axis represents the significant enrichment counts of these terms, while the Y-axis represents the corresponding terms of the target genes.

To clarify the oxidative stress mechanism of GXV in treating VR, we conducted GO and KEGG functional enrichment analysis for 206 shared targets. GO enrichment items were classified into three functional groups: molecular function (Figure 5A), biological process (Figure 5B), and cellular component (Figure 5C). The results indicated that numerous molecular functions were involved in enzyme binding, protein homodimerization activity, drug binding, protein binding, protein heterodimerization activity, identical protein binding, transcription factor binding, ubiquitin-protein ligase binding, type I transforming growth factor beta receptor binding, and transforming growth factor beta receptor, pathway-specific cytoplasmic mediator activity (Figure 5A). The results indicated that numerous biological processes were related to the treatment of VR, including response to the drug, response to hypoxia, aging, negative regulation of the apoptotic process, positive regulation of cell proliferation, positive regulation of transcription from RNA polymerase II promoter, positive regulation of transcription, DNA-templated, positive regulation of smooth muscle cell proliferation, transforming growth factor beta receptor signaling pathway, and cell proliferation (Figure 5B). The results indicated that numerous cellular components were involved in the plasma membrane, cytosol, membrane raft, extracellular space, an integral component of the plasma membrane, cell surface, transcription factor complex, SMAD protein complex, receptor complex, and nucleoplasm (Figure 5C). The top 20 enriched KEGG pathways for the 206 shared targets are shown in Figure 5D. Among these pathways, the HIF-1 signaling pathway, FoxO signaling pathway, neuroactive ligand-receptor interaction, calcium-signaling pathway, PI3K-Akt-signaling pathway, cell cycle, and TGF-β signaling pathway were involved in the development and pathogenesis of VR. In a word, these enrichment findings support the potential pharmacological mechanism of GXV in the treatment of VR. Importantly, the “two GXV pathways” (TGF-β signaling pathway and apoptosis pathway) we obtained before (17) have been verified here. We reconstructed the PPI network diagram of the targets related to the “two GXV pathways” and visualized the pathways (Supplementary Figure S3).

**Figure 6:**
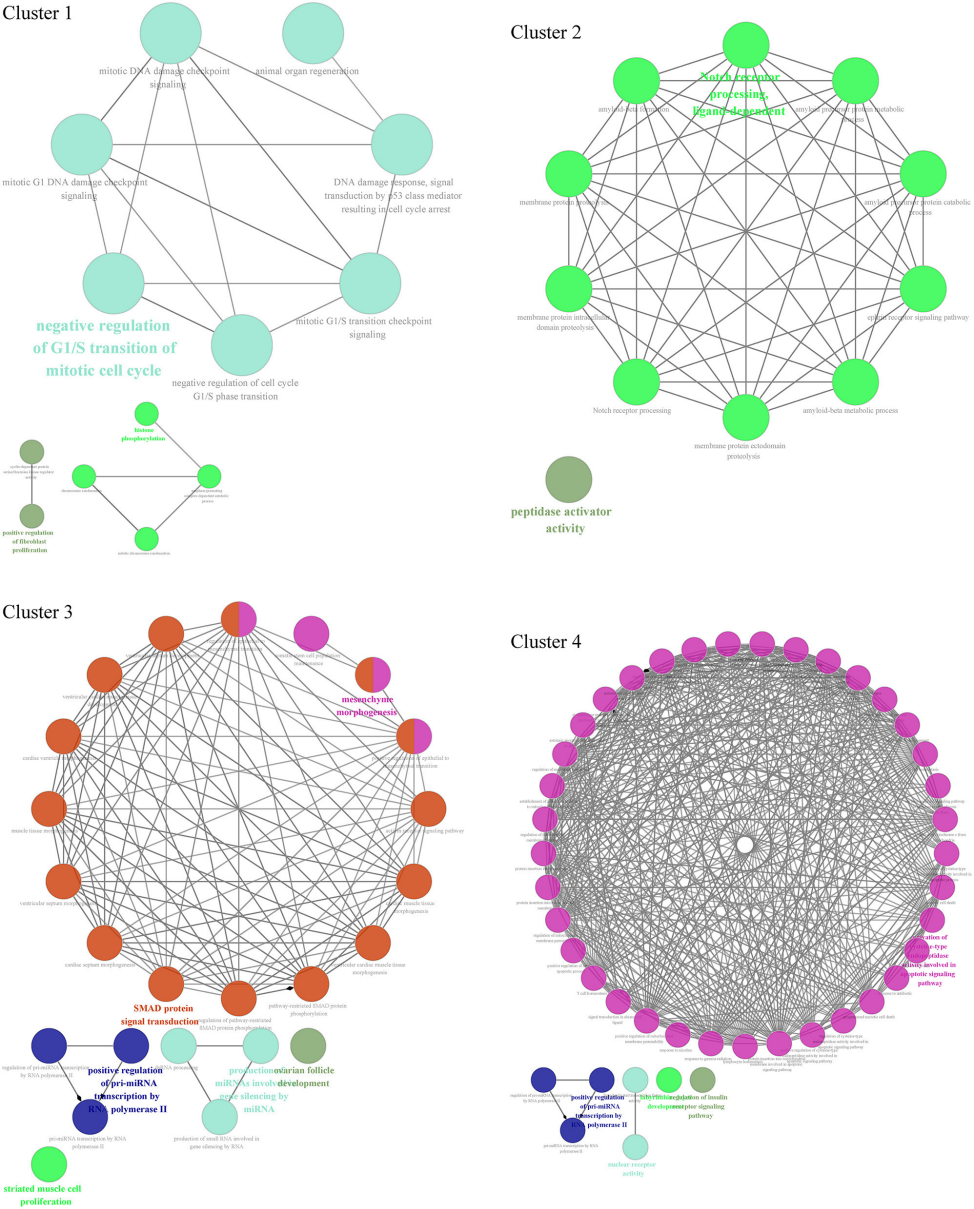
Functional enrichment analysis of 4 cluster networks divided from the PPI network.

Through GO biological process enrichment of 4 cluster networks from the PPI network, we found that cluster 1 enriched in negative regulation of G1/S transition of the mitotic cell cycle, positive regulation of fibroblast proliferation, and histone phosphorylation; cluster 2 enriched in Notch receptor processing and peptidase activator activity; cluster 3 enriched in SMAD protein signal transduction, mesenchyme morphogenesis, striated muscle cell proliferation, positive regulation of pri-miRNA transcription by RNA polymerase II, and production of miRNAs involved in gene silencing by miRNA; and cluster 4 enriched in activation of cysteine-type endopeptidase activity involved in the apoptotic signaling pathway, positive regulation of pri-miRNA transcription by RNA polymerase II, nuclear receptor activity, and regulation of insulin receptor signaling pathway (Figure 6, Supplementary Table S1).

### Computational Validation

**Figure 7:**
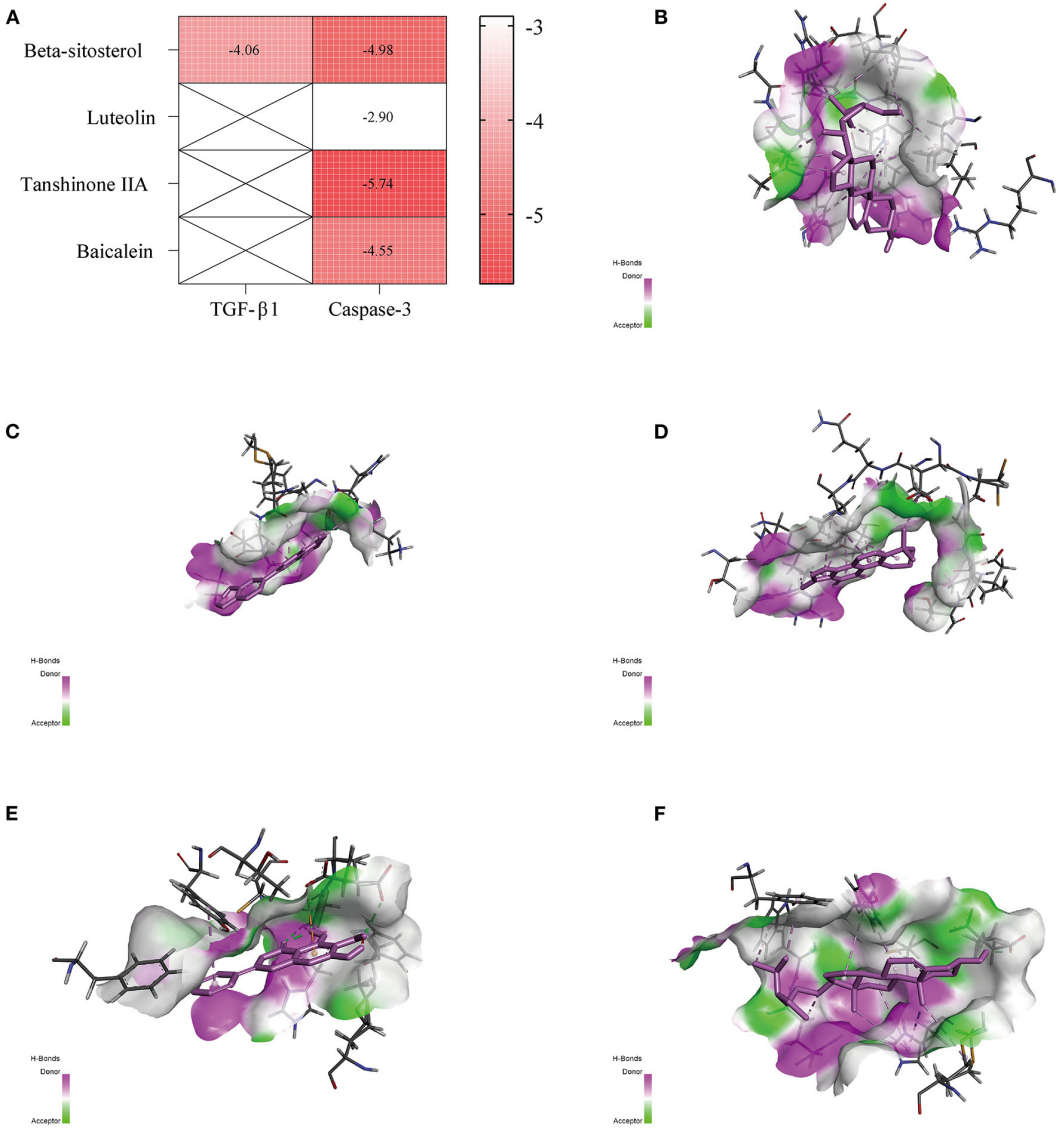
Molecular docking. **(A)** Heatmap of binding energy. **(B)** Transforming growth factor (TGF)-β1 and Beta-Sitosterol. **(C)** Caspase-3 and Luteolin. **(D)** Caspase-3 and Tanshinone IIA. **(E)** Caspase-3 and Baicalein. **(F)** Caspase-3 and Beta-Sitosterol.

The PDB entry codes for TGF-β1 and Caspase-3 are 6P7J (37) and 5I9B (38), respectively, and the PubChem IDs of Beta-Sitosterol, Luteolin, Tanshinone IIA, and Baicalein are 222284, 5280445, 164676, and 5281605, respectively. The compounds from GXV likely interacted strongly with the identified key targets (Figure 7A). The three-dimensional structural diagrams of molecular docking are shown in Figures 7B–F.

### Experimental Validation

**Figure 8:**
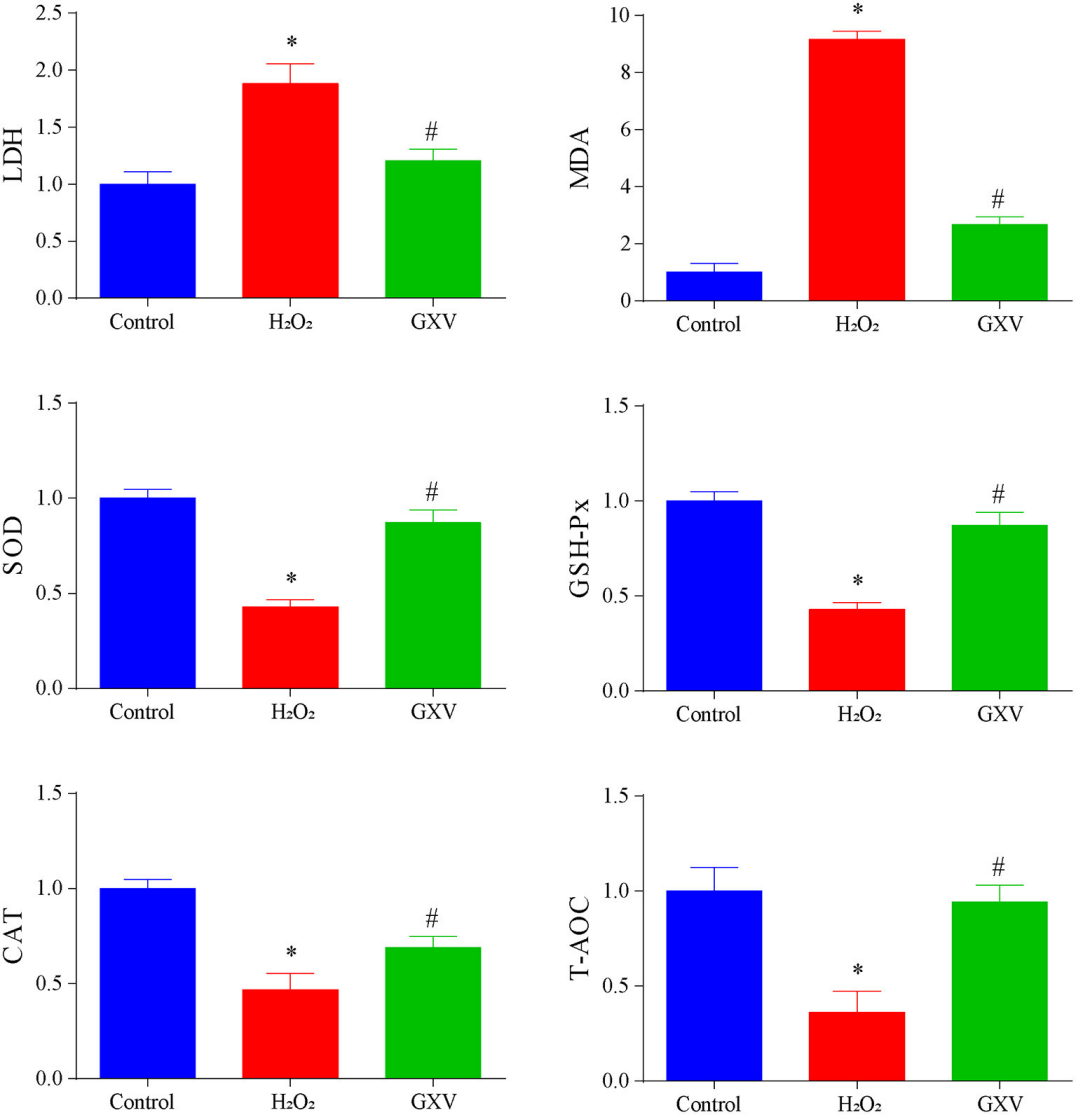
Guanxin V (GXV) alleviates H_2_O_2_-induced oxidative stress. There were at least 3 independent biological replicates in each group of each experiment. *P < 0.05 compared with the Control group, ^#^*P* < 0.05 compared with the H_2_O_2_ group.

H9c2(2-1) cells incubated with 300 μM H_2_O_2_ had increased LDH and MDA and decreased SOD, GSH-Px, CAT, and T-AOC (Figure 8) indicating that the level of oxidative stress was elevated. Administration of GXV could reverse the elevated oxidative stress (Figure 8). Together, we concluded that GXV could consider as an antioxidant.

## Discussion

VR is an important factor leading to the poor prognosis of many cardiovascular diseases in the middle and late stages (39). Therefore, delaying VR is of great significance for improving the quality of life. Our previous studies have shown that GXV has great potential for delaying or even reversing VR (5, 6), here, we aim to detect the oxidative stress-related mechanism of GXV in treating VR through virtual screening and network pharmacology integration strategy and molecular docking, and robust experiments verification strategy to provide evidence of traditional Chinese medicine for VR.

Different from the previous method of obtaining the effective ingredients and targets of drugs directly from the database (25), we identified the effective components of GXV by UPLC-Q-TOF/HRMS E and then obtained the corresponding targets through its structure as a supplement, which can well avoid the bias of target selection. After screening the related datasets, we identified 251 GXV-related targets, 11,425 VR-related targets, and 9,727 oxidative stress-related targets, respectively. Then, we converted these targets data to the G-C-T-V network. From the established G-C-T-V network, we identified several key components and targets through multiple centrality assessments. Luteolin, a widely distributed flavonoid found in many herbal extracts (12), is known to be a potent antioxidant and is demonstrated to have protective actions against Ang II-induced VR, which could be mediated through attenuation of oxidative stress (40). Meanwhile, luteolin improved monocrotaline-induced right VR at least partly through suppressing HIPPO-YAP/PI3K/AKT signaling pathway (41). Tanshinone IIA may alleviate VR in rats by reducing oxidative stress, inflammatory response, and cardiomyocyte apoptosis (42, 43), and enhanced autophagy (44) *via* the inhibition of TLR4/MyD88/NF-κB signaling pathway (45) and activation of SIRT1 signaling pathway (43). Ongoing STAMP-REMODELING trial, a randomized controlled trial, will provide important clinical evidence on the efficacy of Tanshinone IIA in patients with STEMI that might significantly reduce adverse left VR and potentially improve clinical outcomes (46). Baicalein can significantly ameliorate Ang II-induced VR *via* the inhibition of inflammation, oxidative stress, and multiple signaling pathways (AKT/mTOR, ERK1/2, NF-κB, and calcineurin) (47), and monocrotaline-induced vascular remodeling *via* the MAPK and NF-κB pathways (48) and Akt/Erk1/2/GSK3β/β-catenin/ET-1/ET_A_R signaling (49). Besides, we used the shared targets to build the PPI network and simplified the 4 cluster networks. The GO biological processes were enriched proliferation, apoptosis, and TGF-β, which are consistent with our previous research results (6, 17). We also enriched TGF-β signaling pathway and apoptosis pathway that were considered “two GXV pathways” in our previous study (17), in KEGG functional analysis. The enrichment analysis of 4 cluster networks also focused on various biological processes related to VR, which reflects the mechanism related to oxidative stress of GXV in the treatment of VR. More importantly, we computationally validated the interaction between components from GXV and shared targets, and experimentally validated that GXV can indeed alleviate oxidative stress.

There are some limitations that should be considered in our further study. First, traditional Chinese medicine generally has the characteristics of multitarget and multieffect, which means that it is not enough to determine only two pathways, although this is confirmed by our previous research results. Moreover, although we verified the results obtained from virtual screening and network pharmacology through molecular docking and solid experiments, more experiments are still required to further verify our reliable findings. Finally, some important targets of GXV, VR, and oxidative stress might be ignored and missed that is the inevitable bias of network pharmacology (22), so we not only enriched our components acquisition with UPLC-Q-TOF/HRMS E, but also searched as many available databases as possible.

## Conclusions

Our study predicts the targets of the synergistic pharmacological mechanism of GXV and explores the potential mechanism involved in alleviating and even reversing VR through integrating virtual screening and network pharmacology strategy and molecular docking and experimental validation, which provide a complementary and alternative medication for VR.

## Data Availability Statement

The datasets presented in this study can be found in online repositories. The names of the repository/repositories and accession number(s) can be found in the article/Supplementary Material.

## Author Contributions

BL and NG conceived, designed, and planned the study. BL and YL acquired and analyzed the data. BL and RL completed the *in vitro* experiments. All authors interpreted the results. BL drafted the manuscript. NG contributed to the critical revision of the manuscript. All authors read and approved the final manuscript.

## Funding

This work was partly funded by Research and Practice Innovation Plan for Postgraduates of Jiangsu, China [KYCX21_1641], National Natural Science Foundation of China [81774229], Jiangsu Leading Talent Project of Traditional Chinese Medicine [Jiangsu TCM 2018 No.4], and Jiangsu Universities Nursing Advantage Discipline Project [2019YSHL095].

## Conflict of Interest

The authors declare that the research was conducted in the absence of any commercial or financial relationships that could be construed as a potential conflict of interest.

## Publisher's Note

All claims expressed in this article are solely those of the authors and do not necessarily represent those of their affiliated organizations, or those of the publisher, the editors and the reviewers. Any product that may be evaluated in this article, or claim that may be made by its manufacturer, is not guaranteed or endorsed by the publisher.

## Supplementary Material

Supplementary Table S1 Functional enrichment analysis of 4 cluster networks divided from the protein-protein interaction (PPI) network.

Supplementary Figure S1 Venn diagram.

Supplementary Figure S2 The PPI network with all interactions.

Supplementary Figure S3 PPI networks and Kyoto Encyclopedia of Genes and Genomes (KEGG) pathways of “two GXV pathways”. **(A)** The PPI network with all interactions of “two GXV pathways”. The red nodes represent the transforming growth factor (TGF)-β signaling pathway, and the blue nodes represent apoptosis. **(B)** The PPI network with known interactions comes from experimentally determined evidence. The red nodes represent the TGF-β signaling pathway, and the blue nodes represent apoptosis. **(C)** KEGG pathway map of TGF-β signaling pathway. The red pentagrams indicate the genes shared by GXV and ventricular remodeling. **(D)** KEGG pathway map of apoptosis. The red pentagrams indicate the shared targets.
