# Supplementary figures and images for: Guanxin V Acts as an Antioxidant in Ventricular Remodeling
Source: Front Cardiovasc Med. 2022 Jan 4;8:778005. doi: 10.3389/fcvm.2021.778005 (PMC8764413; doi:10.3389/fcvm.2021.778005)

GXV

oxidative stress

13

25

3002

206

7

6494

4718

VR

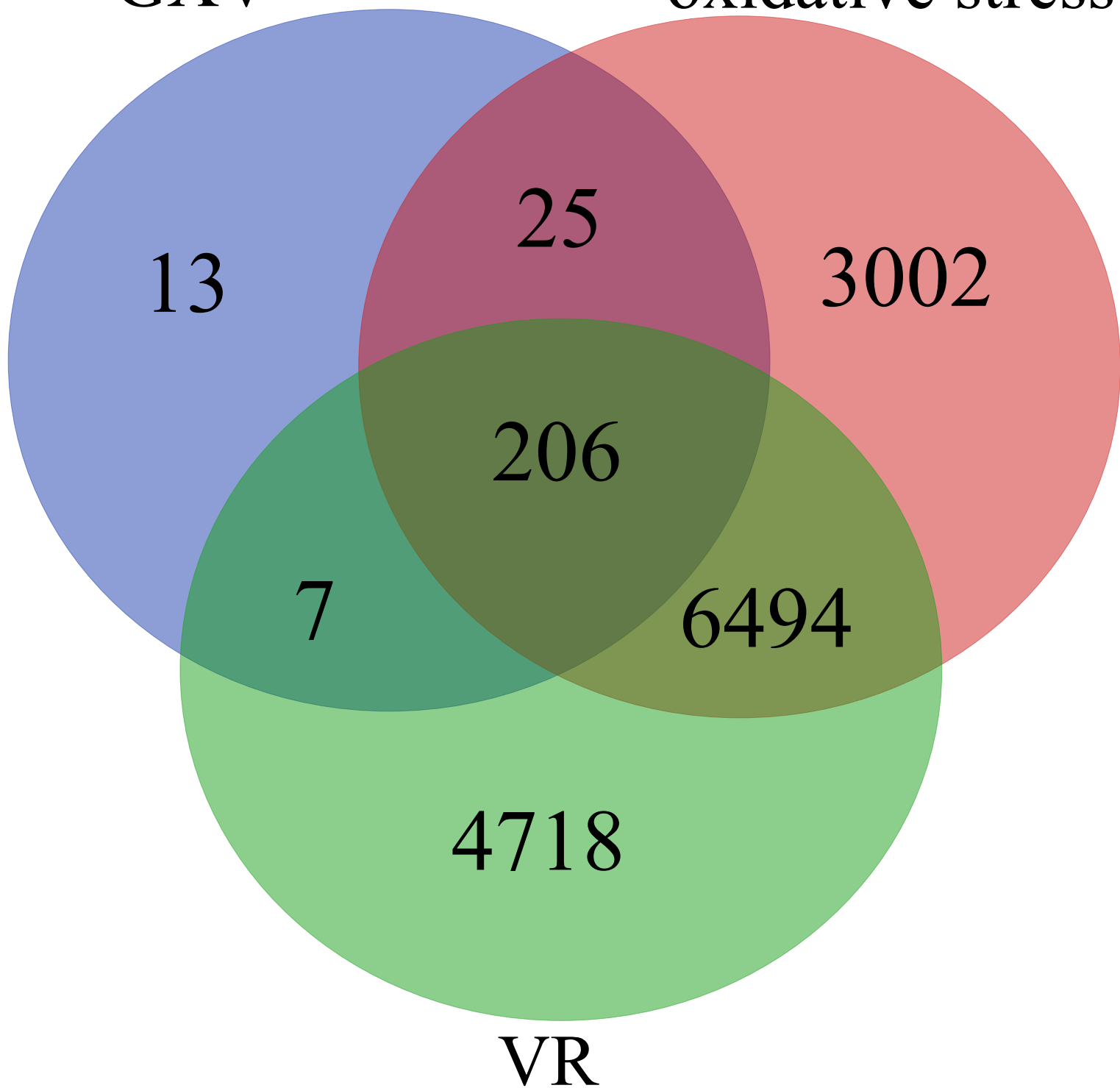

Supplement: Supplementary Figure S1 — Venn diagram. [file Data_Sheet_1.PDF]

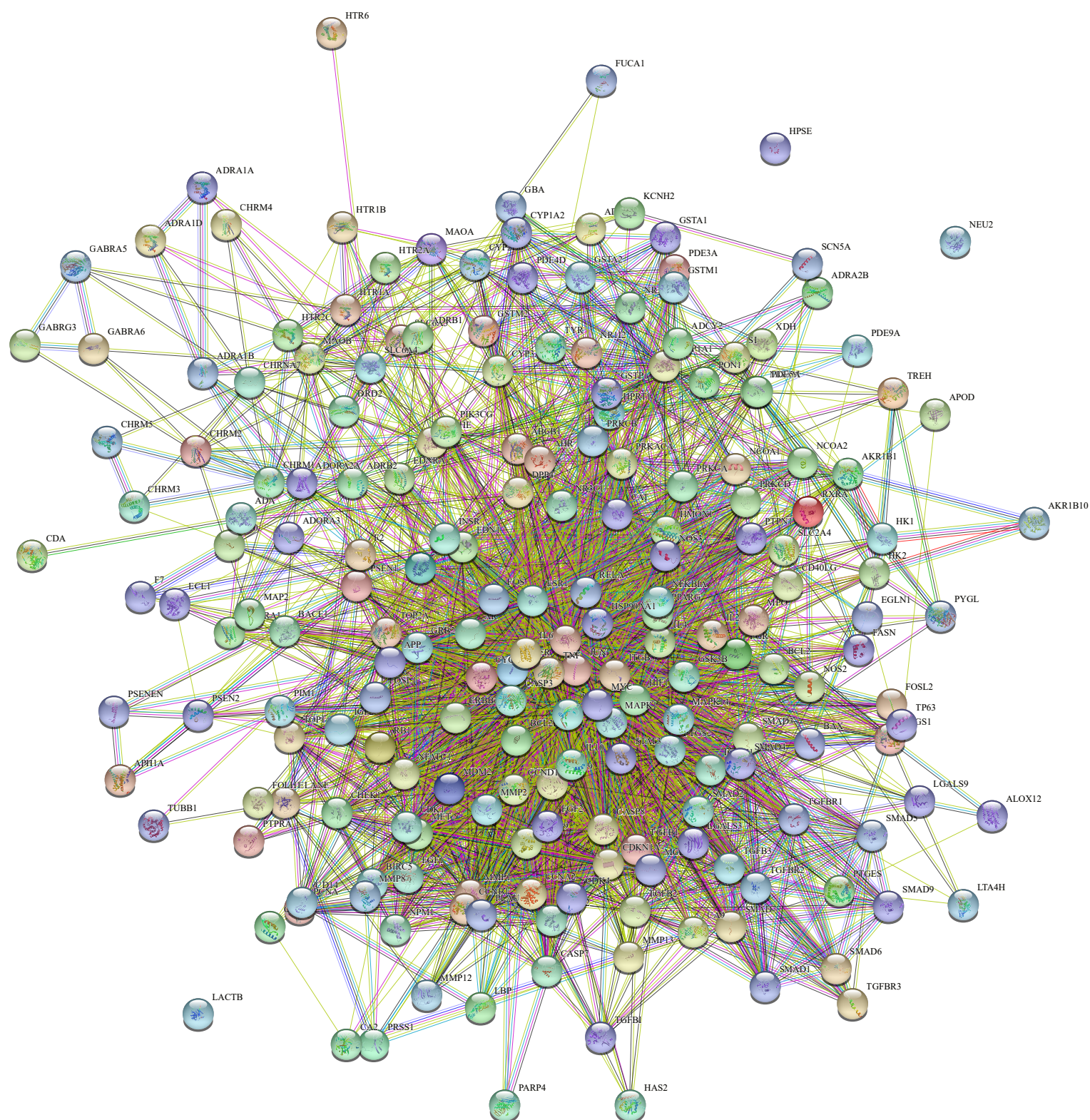

Supplement: Supplementary Figure S2 — The PPI network with all interactions. [file Data_Sheet_2.PDF]
