# Supplementary material for: Guanxin V Acts as an Antioxidant in Ventricular Remodeling
Source: Front Cardiovasc Med. 2022 Jan 4;8:778005. doi: 10.3389/fcvm.2021.778005 (PMC8764413; doi:10.3389/fcvm.2021.778005)
Supplement: Supplementary Figure S3 — PPI networks and Kyoto Encyclopedia of Genes and Genomes (KEGG) pathways of “two GXV pathways”. (A) The PPI network with all interactions of “two GXV pathways”. The red nodes represent the transforming growth factor (TGF)-β signaling pathway, and the blue nodes represent apoptosis. (B) The PPI network with known interactions comes from experimentally determined evidence. The red nodes represent the TGF-β signaling pathway, and the blue nodes represent apoptosis. (C) KEGG pathway map of TGF-β signaling pathway. The red pentagrams indicate the genes shared by GXV and ventricular remodeling. (D) KEGG pathway map of apoptosis. The red pentagrams indicate the shared targets. [file Data_Sheet_3.PDF]

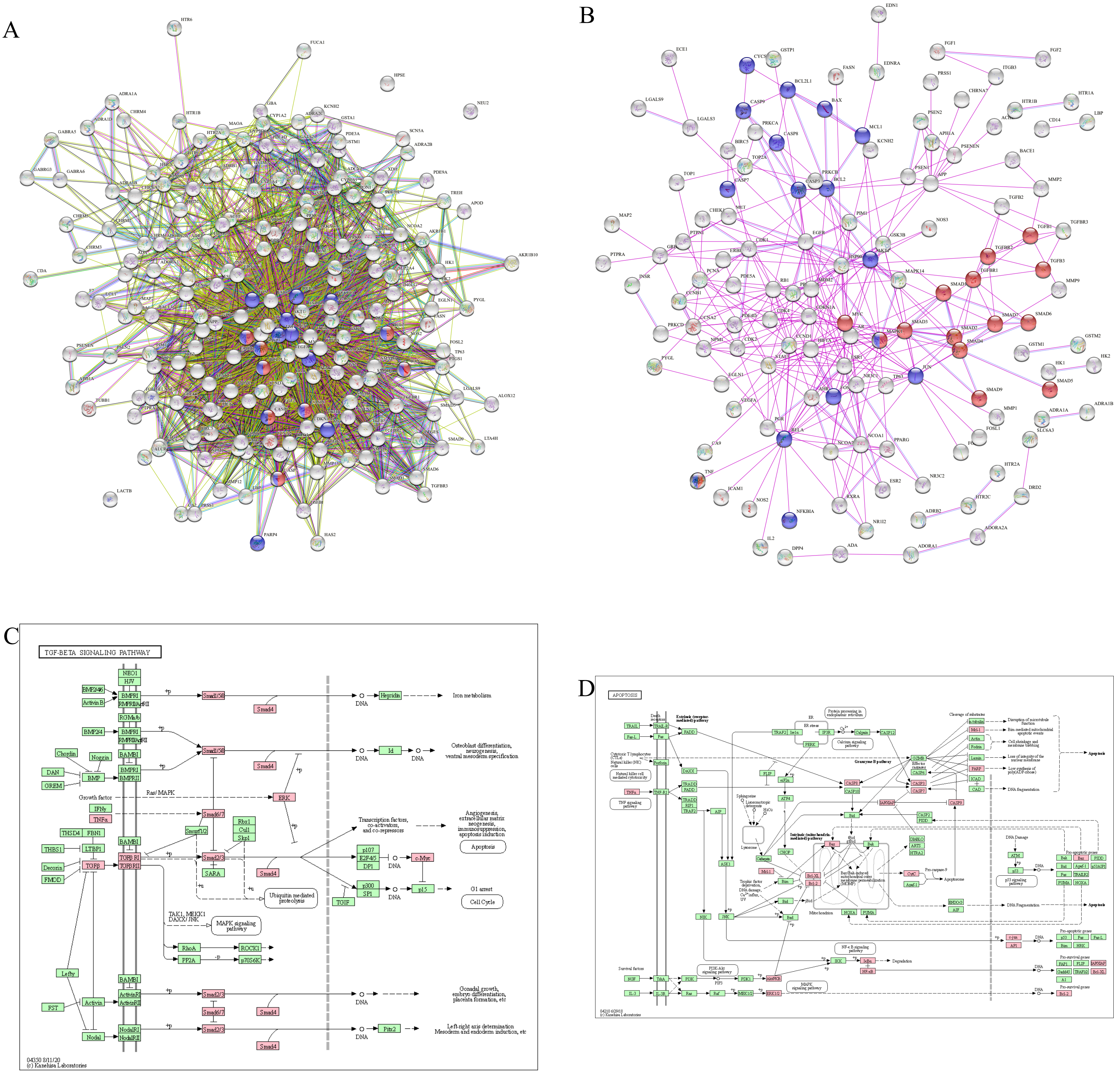

A
B
C
TGF-BETA SIGNALING PATHWAY
D
APOPTOSIS
